# The Manipulation of Pace within Endurance Sport

**DOI:** 10.3389/fphys.2017.00102

**Published:** 2017-02-27

**Authors:** Sabrina Skorski, Chris R. Abbiss

**Affiliations:** ^1^Institute of Sports and Preventive Medicine, Saarland UniversitySaarbrücken, Germany; ^2^Centre for Exercise and Sports Science Research, School of Exercise and Health Sciences, Edith Cowan UniversityJoondalup, WA, Australia

**Keywords:** pacing factors, energy expenditure, fatigue, kinematic, exercise environment

## Abstract

In any athletic event, the ability to appropriately distribute energy is essential to prevent premature fatigue prior to the completion of the event. In sport science literature this is termed “*pacing*.” Within the past decade, research aiming to better understand the underlying mechanisms influencing the selection of an athlete's pacing during exercise has dramatically increased. It is suggested that pacing is a combination of anticipation, knowledge of the end-point, prior experience and sensory feedback. In order to better understand the role each of these factors have in the regulation of pace, studies have often manipulated various conditions known to influence performance such as the feedback provided to participants, the starting strategy or environmental conditions. As with all research there are several factors that should be considered in the interpretation of results from these studies. Thus, this review aims at discussing the pacing literature examining the manipulation of: (i) energy expenditure and pacing strategies, (ii) kinematics or biomechanics, (iii) exercise environment, and (iv) fatigue development.

## Introduction

Any athletic event inevitably has a beginning and an endpoint. In order to reach this endpoint in the fastest possible time, athletes need to appropriately distribute their energy expenditure, in a way that all available energetic resources are used but not so early so as to experience premature fatigue prior to the finish line (St. Clair Gibson et al., 2006). In sport science literature this has been termed as “*pacing*,” “*pacing strategy*,” “*pacing profile*” and/or “*pacing pattern*.” This regulation of speed, power or energy expenditure throughout an exercise task is extremely important in the optimisation of performance. Based on current research, pacing appears to be regulated by complex relationships between the brain and other physiological systems (St. Clair Gibson and Noakes, 2004; Noakes et al., 2005; Abbiss and Laursen, 2008). Several models have been proposed to explain this phenomena including: the teleoanticipatory theory (Ulmer, 1996; St. Clair Gibson and Noakes, 2004), the central governor model (Noakes et al., 2001), the perception based model (Tucker, 2009), the pacing awareness model (Edwards and Polman, 2013) and the psychobiological model (Marcora, 2010; Pageaux, 2014).

Since our last review on athletic pacing (Abbiss and Laursen, 2008) research examining physiological, psychological and environmental factors influencing pacing during exercise has dramatically increased. Much of this research has: (i) observed the pacing profiles adopted by athletes in competition (Abbiss and Laursen, 2008), (ii) examined the effects of directly manipulating pacing on performance (Foster et al., 1993; Aisbett et al., 2009a; Skorski et al., 2014), or (iii) examined the effects of manipulating feedback (Albertus et al., 2005; Mauger et al., 2009; Faulkner et al., 2011; Castle et al., 2012), task demands or environmental conditions (Clark et al., 2007; Abbiss et al., 2010; Peiffer and Abbiss, 2011) on pacing and performance. Such research has been instrumental in improving our understanding of how humans regulate energy expenditure and the “optimal” pacing strategies to adopt during various exercise tasks. However, as with all research there are several factors that should be considered in the interpretation of results from these studies. This manuscript will discuss these considerations in the context of pacing. Specifically this review will discuss factors important to the interpretation of pacing literature examining the manipulation of: (i) energy expenditure or pacing strategies, (ii) kinematics or biomechanics, (iii) exercise environment, and/or (iv) fatigue development.

## Manipulation of energy expenditure or pacing

Technological advancements have made it easier to quantify both mechanical power output and resistive forces experience during exercise and as a result it has been possible to mathematically model optimal pacing profiles by minimizing the time required to complete a given amount of work. Such modeling typically indicates that exercise performance can be optimized if athletes produce greater power output during periods of high external resistance (i.e., when accelerating, traveling into a headwind or against gravity) and lower power outputs produced during periods of lower resistance (Swain, 1997; Atkinson et al., 2007; Wells and Marwood, 2016). However, the degree to which an athlete might be able to vary exercise intensity, in order to compensate for high resistive forces, is dictated by their psychophysiological capabilities (Atkinson et al., 2007). Indeed, most theoretical models highlight cognitive and physiological characteristics as key variables that dictate the regulation of pacing during exercise (St. Gibson et al., 2006; Pageaux, 2014; Renfree et al., 2014).

Several studies aimed at better understanding optimal pacing profiles have deliberately forced participants to adopt specific pacing strategies in order to determine, if such pacing profiles improve performance above theoretically submaximal strategies (Aisbett et al., 2009a,b) and if athletes are able to adhere to the required power outputs (Thomas et al., 2013). An important consideration with regards to these studies is that forcing athletes to adopt a given workload, even if only for a short period of an event, could in itself influence overall performance. Indeed, choice or the anticipation of the opportunity for choice has been associated with increased activity of brain regions directly involved in reward processing (Fujiwara et al., 2013; Lewthwaite et al., 2015) and has therefore been considered as an important regulator e.g., for motor learning (Lewthwaite et al., 2015). Thus, forcing athletes to adopt a given pacing profile might remove self-choice, giving them less control over the situation and influence performance and motivation. It has also been shown that compared with self-paced exercise, externally controlling exercise intensity alters attentional focus, resulting in the predominance of reactive, rather than proactive cognitive control (Brick et al., 2016). Interestingly, a reduction in the ability to control exercise intensity does not only influence cognitive function but also results in greater physiological stress and reduced exercise capacity, when compared to a self-paced exercise task (Billat et al., 2006; Lander et al., 2009). For instance, Thomas et al. (2013) found that several participants were unable to complete the same amount of work during an externally controlled, even paced cycling trial, when compared with prior self-paced 20 km cycling trials. Conversely, several studies have also observed lower physiological stress during externally controlled trials (Zadow et al., 2015; Brick et al., 2016), when compared with matched work self-paced trials, possibly as a result of reduced cognitive load (Garcin et al., 2008; Lander et al., 2009). However, despite of lower physiological strain perceptual responses (i.e., RPE) were similar (Thomas et al., 2013) or even higher (Lander et al., 2009) in externally controlled trials. Thus, the ability to voluntarily fluctuate power output in accordance with sensations of fatigue during the exercise bout seems to be an important factor in the regulation of pace (Lander et al., 2009). Enforced or externally paced strategies, force the athlete to abandon their pacing pattern and minimize opportunities for self-managing fatigue (Lander et al., 2009).

The reason for inconsistencies with regards to the effects of externally controlling pace, within the literature, is not clear. However, it should also be noted that within the current pacing literature various methods have been used in order to enforce specific pacing strategies. Such methods have included ergometers set to automatically regulate intensity regardless of stroke rate or cycling cadence (Abbiss et al., 2009), or requirements of athletes to maintain a given intensity based on visual (Zadow et al., 2015) or audio feedback (Thompson et al., 2003; Skorski et al., 2014). Furthermore, some studies used simulated competitors (i.e., avatars) to examine the effects of their presence on self-selected pacing strategies (Shei et al., 2016). It should be noted that each of these methods not only differ in the mechanisms by which external pacing is controlled, but also in the feedback provided to participants. Given that the cognitive demand and attentional focus is likely to differ between each of these methods caution should be taken comparing results across studies or in the implementation of such findings into the field. It is of note that the current literature which has enforced a specific pacing condition, in order to approximate mathematical models or better understand optimal pacing strategies, has typically only performed a single trial in each pacing condition. While research has shown consistent and moderate reliability in cycling time trials with an enforced starting pace (first quarter of 5 min cycling time trials, Aisbett et al., 2015), we are unaware of any studies that have deliberately and systematically trained an athlete's pacing profile. Such research might be meaningful since it has been shown that both training (Kennedy and Bell, 2003) and heat acclimation (Racinais et al., 2015) results in the adoption of a more even pacing profile during an endurance task, which is theoretically optimal based on mathematical modeling and better approximates the profile adopted by well-trained or elite athlete (Wilberg and Pratt, 1988).

## Manipulation of kinematics and biomechanics

To date, research examining the effects of manipulating kinematic and/or biomechanical variables on pacing and performance is somewhat limited. This is surprising, considering that these variables are often manipulated in sport science practice in an attempt to improve force/power development, prevent fatigue, reduce fluid resistance and ultimately improve performance (Overton, 2013). Moreover, it is plausible that athletes change movement kinematics in order to prevent premature fatigue or vice versa that the fatigue state changes an athletes movement patterns. For example, it has previously been found that during constant-pace middle-distance (~5–6 min) cycling to task failure, cyclists increase trunk flexion as peripheral fatigue (i.e., decrease in force or power resulting from changes beyond the neuromuscular junction) develops, presumably in order to increase muscle recruitment and torque production at the hip and knee (Overton, 2013). Additionally, within this study it was also found that significant changes in the angle orientation of the trunk, hip and knee in the coronal plane throughout the pedal cycle were observed from 80 to 100% of task failure and paralleled increases in EMG amplitude (Overton, 2013). Further, vertical leg stiffness decreases with increasing fatigue which results in a decrease in stride frequency during sprint running (Morin et al., 2006). In this regard, world-class runners show a larger decease in stride frequency within the last 50 m of a 400 m running race when compared to recreational athletes (Hanon and Gajer, 2009). As velocity decreases in the last part of a 400 m run, stride length and stride frequency also decrease. The decrease in stride length already occurs between 200 and 300 m, whereas stride frequency can mostly be attributed to the last 50 m (Hanon and Gajer, 2009). This decrease in stride frequency might be due to a greater stride length earlier in the race which increases ground force production and thus fatigue development (Mero et al., 1992; Hanon and Gajer, 2009). These findings are important as they highlight that fatigue development, kinematics and biomechanics are not consistent throughout an exercise task, which influences the quantification of optimal pacing using mathematical modeling.

To date, few studies have deliberately manipulated movement kinematics in order to examine the influence on self-selected pacing strategies. However, studies have examined the influence of manipulating various biomechanical/kinematic variables on power/force production and fatigue development. Indeed, it is often found that performance is reduced when forcing cyclists to adopt a pedaling rate that differ considerably from self-selected cadences, even if such cadences are more economical or reduce neuromuscular fatigue development (Abbiss et al., 2009). It has also been shown that instructing cyclists to maintain consistent trunk and hip joint kinematics during a fatiguing bout of cycling reduces the time to task failure in some but not all athletes. These results indicate that restricting kinematic variation that normally occurs, presumably in order to overcome fatigue development, might compromise performance (Overton, 2013). The reason time to task failure is reduced when joint kinematics are held consistent is currently unclear but may be due to an athlete's inability to manage fatigue by slightly changing movement kinematics (Overton, 2013) or associated the restriction of self-choice, thereby altering motivation (Fujiwara et al., 2013; Lewthwaite et al., 2015). Regardless, such findings are important in the mathematical modeling of optimal pacing since kinematics and fluid resistance is not consistent during the exercise task.

A change in kinematics is likely to be even more crucial in water-based sports like rowing or swimming where resistive forces are higher compared to land-based sports (Smith et al., 2002; Maglischo, 2003). Swimmers usually increase speed by a combination of increasing stroke length and/or stroke rate throughout the event (Smith et al., 2002). Hence manipulating one or both factors might be crucial for swim efficacy, economy and thus performance (Aspenes and Karlsen, 2012). For instance, Swaine and Reilly (1983) found significant changes in oxygen uptake and minute ventilation when stroke rate was manipulated during high-intensity front-crawl swimming on a swim bench. A later study manipulated stroke rate during 200 m breaststroke swimming (Thompson et al., 2003). Using a programmable audible pacing device swimmers were paced at 91, 95, 100, and 107% of their average stroke rate in a previous self-paced trial, yet no statistical significance between trials was observed in performance times, physiological responses (heart rate, blood lactate, oxygen-uptake) and rating of perceived exertion (Thompson et al., 2003). However, when athletes were forced to increase their pace by 2% they swam the 200 m with a significantly higher stroke rate (Thompson et al., 2004). Thus, even though research looking into possible effects of manipulation kinematics and/or biomechanics on pacing is lacking, there is a strong rationale to assume that such enforced changes might alter fluid resistance, fatigue development and optimal pacing during exercise.

## Manipulation of exercise environment

In order to further our understanding of the mechanisms important in the regulation of exercise intensity a large number of studies have observed the self-selected pacing profiles of athletes in a range of environmental conditions but particularly hypoxic or hot conditions. While exercise capacity is reduced in hypoxia, few studies to date have observed drastic changes in pacing during exercise in hypoxia. Conversely, a recent meta-analysis demonstrated that during prolonged exercise in the heat a clear decrease in exercise intensity from commencement of the trial is typically observed (Davies et al., 2016). The authors explain this by the nature of heat interventions, as a marked change in room temperature is easier to identify by participants compared to a change in oxygen content (Davies et al., 2016). It is therefore likely that adjustments in power output or speed are made sooner in the heat trials due to earlier changes in afferent feedback (Davies et al., 2016). Conversely, maximal oxidative capacity and power output is reduced upon exposure to a hypoxic environment compared to normoxia (Davies et al., 2016). Interestingly though, the meta-analysis showed that power output in the starting section of the hypoxia trials was not significantly different to normoxia, which indicates a delay in the adjustment of the pacing pattern in hypoxia (Davies et al., 2016). Such findings are important since they highlight that physiological stress during exercise is not always constant and as a result the optimal pace to adopt is dynamic and might differ to those predicted in current mathematical models (i.e., even pacing).

It is important to note that altering environmental conditions might influence pacing through effecting not only physiological but also psychological state/function. Indeed, the above mentioned alterations might not only influence muscle oxygen delivery and peripheral fatigue but also influence central motor drive to the exercising skeletal musculature (Davies et al., 2016). It should also be noted that several strategies of altering the exercise environment are likely to influence pacing with greatest effect on cognitive/psychological, rather than physiological function. For instance, it has been shown that inaccurate feedback (Wilson et al., 2012) or the type of feedback (distance vs. duration) (Abbiss et al., 2016) can alter self-selected pacing. The effects of such manipulations on pacing could be the result of effects on motivation, task engagement, attentional focus, and range of other factors believed to be important to pacing during exercise. While the precise mechanisms are beyond the scope of this manuscript, it should be noted that as with physiological stress, one's cognitive state is dynamic and does not necessarily increase linear with exercise duration (de Koning et al., 2011; Wilson et al., 2012). Consequently, understanding the kinetics of central, peripheral and mental fatigue development during exercise is likely to be important in the determination of optimal pacing strategies.

## Manipulation of fatigue development

It is well accepted that central (Meeusen et al., 2006; Taylor et al., 2016), peripheral (Marcora and Staiano, 2010; de Morree and Marcora, 2013; Taylor et al., 2016) and mental fatigue (Marcora et al., 2009; Pageaux et al., 2015) development reduces exercise capacity. However, the influence of such fatigue on pacing during exercise is not entirely clear. As such, recent research has examined the effects of systematically manipulating fatigue development in various regions of the body on self-selected pacing during exercise. Over decades researchers have been arguing on the different origins of fatigue, especially the difference between central and peripheral fatigue. As recently outlined a reason for this dilemma might be the inability of current terminology to accommodate the scope of conditions ascribed to fatigue (Enoka and Duchateau, 2016). Defining the different terms used in the following paragraph in regards to fatigue would extend the scope of this review and have been published elsewhere (St. Clair Gibson and Noakes, 2004; Abbiss and Laursen, 2005; Noakes et al., 2005; Meeusen et al., 2006; Enoka and Duchateau, 2016; Taylor et al., 2016).

### Effects of pre-exercise peripheral fatigue

Amann and Dempsey (2008) previously described the effects of locomotor muscle fatigue on overall 5-km time trial performance. In this study it was found that compared to a control condition, time to completion as mean power output during a 5 km cycling time trial was reduced following constant-workload cycling until exhaustion (Amann and Dempsey, 2008). Pre-exercise (peripheral) fatigue levels were assessed via changes in potential twitch force of the vastus lateralis, vastus medialis, and rectus femoris. Potential twitch force was significantly reduced after the constant-workload cycling indicating substantial levels of locomotor muscle fatigue (Amann and Dempsey, 2008). Time to completion was reduced by 2 to 6% and mean power output by 4 to 14% compared to the non-fatigued control condition. Given that power output rose to almost identical levels in the final 200 m of each trial, despite different levels of pre-exercise fatigue, it was speculated that during the trial subjects adopted a more conservative exercise intensity to avoid further accumulation of peripheral fatigue before the end of the trial (Amann and Dempsey, 2008). As such, the authors of this study suggested, that the development of locomotor muscle fatigue an important influence on central motor drive and consequently can compromise exercise performance (Amann and Dempsey, 2008).

Interestingly, a recently published study described the effect of a pre-exercise eccentric fatiguing protocol (100 drop jumps) on pacing in a 15-min cycling trial conducted 30 min after (de Morree and Marcora, 2013). There was no difference in pacing between conditions, even though overall performance was worse in the fatigued trial (de Morree and Marcora, 2013). Nonetheless, participants were again able to increase power output in the last 3 min of the fatigued trial resulting in a similar end-spurt as in the control condition (de Morree and Marcora, 2013). The authors suggested that finishing the race is paramount thus participants choose a lower power output for most of the trial. Near the end of the trial, when the risk of not finishing the race is negligible, most participants significantly increase power output in the end spurt (de Morree and Marcora, 2013).

To summarize, it seems that pre-exercising interventions compromising locomotor muscle performance, either metabolically (Amann and Dempsey, 2008) or mechanically (de Morree and Marcora, 2013), impair overall performance but not the pacing pattern. As previously shown pacing seems to be stable under various conditions (Stone et al., 2011; Thomas et al., 2012; Périard and Racinais, 2016). However, in several endurance competitions, athletes have to compete in different races over several days and/or weeks (e.g., stage races, heats, and finals) pushing various physiological systems to exhaustion (Abbiss and Laursen, 2005), which supposedly results in a multifaceted accumulation of fatigue.

### Effects of accumulated fatigue

Since it is believed that our distribution of exercise intensity is regulated to prevent premature fatigue, it is likely that pacing is altered by interventions that result in either different amounts of stored energy before exercise or altered substrate use during exercise (Tucker, 2009). In this regard, a recently published study observed that fatigue induced by a 6 day training intervention impacted pacing at the beginning and end of 40 km cycling time trials (Skorski et al., 2015). The 6 day training period consisted of two cycling sessions a day. The morning sessions consisted of either constant high-intensity endurance training or high-intensity interval training in an alternating manner. In the afternoon sessions, participants had to cycle for 3 h at a moderate-intensity. Participants adopted a parabolic-shaped pattern in both recovered conditions, whereas in the fatigued condition, the pattern was even from the beginning with a greater end-spurt in the last 4 km (slow–fast pacing (Abbiss and Laursen, 2008). The authors of this study hypothesized that training induced fatigue might have a stronger effect on pacing than muscle fatigue *per se* (Skorski et al., 2015). Indeed, whole body fatigue development is multifaceted and influenced by several metabolic, neuromuscular and psychological pathways. Within this study heart rate, blood lactate and respiratory exchange ratio during the time trial were significantly reduced after 6 days of hard training, indicating reduced sympathetic nervous system activity (Skorski et al., 2015). This might lead to disturbed glycolytic energy mobilization and cardiovascular responses (Jeukendrup et al., 1992; Halson and Jeukendrup, 2004; Faude et al., 2009) and a shift of the energy-supplying process in favor of increased fat and decreased carbohydrate use (Jeukendrup et al., 1992). However, after 2 days of recover athletes showed the same pacing pattern as before the training period. Nonetheless, such a shift in substrate utilization might be of practical relevance regarding that in several endurance competitions, athletes have to compete in different races over several days and/or weeks (e.g., stage races, heats, and finals), supposedly leading to a similar accumulation of fatigue. For example, road cyclists compete on 90–100 competition days, comprising 1-d races, 1-wk tour races, and 3-wk tour races (Abbiss and Laursen, 2005). Within each of these races, cyclists might perform different competition requirements (e.g., flat, long stages, time trials, uphill ascents), resulting in exhaustion of various physiological systems (Mujika and Padilla, 2001; Abbiss and Laursen, 2005). Depending on the competition and the stage type, heart rate values range between 51 and 89% of maximum hear rate, with power outputs between 192 and 380 W (Mujika and Padilla, 2001). This leads to high demands of aerobic and anaerobic pathways, which might result in a multifaceted accumulation of fatigue and hence a shift in substrate utilization.

### Effects of mental fatigue

Mental fatigue is a change in psychobiological state (Marcora et al., 2009) which has subjective and objective manifestations including increased resistance against further effort (Meijman, 2000), changes in mood (Hockey, 1983) and feelings of “tiredness” (Martin et al., 2015). Mental fatigue has been shown to reduce exercise capacity during prolonged moderate and high-intensity exercise (Marcora et al., 2009; Brownsberger et al., 2013). However, Martin et al. (2015), recently found that maximal anaerobic performance was not affected by a mentally fatiguing task performed prior to a 3-min all-out cycling test. The authors speculate that this might be due to the type of the task (Martin et al., 2015). In contrast to the aforementioned studies Martin et al. (2015) analyzed the effects of mental fatigue on a short anaerobic cycling task. During short events it appears that an increase in peripheral fatigue development might be associated with an increase in central nervous recruitment to prevent a decrease in performance (Martin et al., 2015). Conversely, prolonged endurance exercise performance might also be influenced by a development of central fatigue resulting in a reduction of voluntary muscle activation (Gandevia, 2001). Indeed, Thomas et al. (2015) recently observed that the contribution of central and peripheral process to fatigue is task-dependent. Shorter, high intensity time trials result in a greater degree of peripheral fatigue, whereas central fatigue has an increased contribution to longer, lower intensity time trials (Thomas et al., 2015). Thus, short-duration maximal anaerobic tasks might be less influenced by alterations in the mental state when compared with the fatigue development during prolonged aerobic endurance exercise (Martin et al., 2015).

Mental fatigue appears to have little influence on physiological factors (e.g., heart rate, oxygen consumption) (Marcora et al., 2009; Pageaux et al., 2015) but often results in greater ratings of perceived exertion when compared to non-fatigue conditions (Marcora et al., 2009; Marcora and Staiano, 2010). As such, it is hypothesized that rating of perceived exertion plays an important role in self-selected pacing which is in accordance with the psychobiological model of endurance performance recently proposed by Marcora (2010) and Pageaux (2014). Indeed, it has been suggested that exercise intensity is regulated based on one's perceived exertion in order to ensure that “catastrophic” or “critical” disturbances to homeostasis do not occur (Noakes et al., 2001; St. Clair Gibson et al., 2006). This is supported by the relatively stable increase in perceived exertion that is typically observed during high-intensity, self-paced exercise (i.e., a time trial) (Noakes, 2008; Cohen et al., 2013). It has been further proposed that the product of the momentary perceived exertion and the fraction of distance remaining (referred to as a hazard score) might provide an indication of changes in intensity during self-paced exercise (de Koning et al., 2011). Importantly, it has also been suggested that one's perception of effort is centrally derived and largely unaffected by peripheral afferent sensory feedback; however, the role of afferent feedback in the regulation of intensity during self-paced exercise has been debated (Marcora, 2009, 2010; Amann and Secher, 2010).

Even though there is an increasing research interest in effects of mental fatigue on endurance performance and pacing it has to be noted that the majority of the published studies generally used artificial and relatively short-term cognitively demanding tasks to induce mental fatigue (e.g., 30 to 90 min Stroop-tests). These tasks might not be directly transferable into athletic or everyday environments, thus research looking into the effects of more practically relevant tasks is needed. Additionally, to the author's knowledge no research, to date, has been published examining the effects of accumulated mental fatigue on performance and/or pacing. Such research, however, is warranted regarding that anecdotal reports from coaches and athletes seem to describe mental fatigue as a phenomenon that is rather developed throughout a season than in acute scenarios.

## Conclusions

Much of the research assessing the accuracy or validity of theoretical and mathematical models of pacing has examined the psychobiological response to directly manipulating the pacing of individuals, and/or examined the influence of manipulating kinematics or biomechanics, exercise environment, and/or fatigue development on self-selected pacing strategies. Given the complexity of how humans regulate pace, it is likely that the manipulation of anyone of these factors has considerable effect on a range of aspects important to task performance. As such, understanding the effects of manipulating such factors will aid in better understanding optimal pacing.

## Author contributions

All authors listed, have made substantial, direct and intellectual contribution to the work, and approved it for publication.

### Conflict of interest statement

The authors declare that the research was conducted in the absence of any commercial or financial relationships that could be construed as a potential conflict of interest.
